# The relationship between real-life social support and Internet addiction among the elderly in China

**DOI:** 10.3389/fpubh.2022.981307

**Published:** 2022-08-26

**Authors:** Yu Jia, Tianyuan Liu, Yang Yang

**Affiliations:** ^1^School of Journalism and Communication, Wuhan University, Wuhan, China; ^2^School of Sociology, Wuhan University, Wuhan, China

**Keywords:** real-life social support, Internet addiction, hopefulness, loneliness, the elderly

## Abstract

Internet addiction among the elderly is a novel issue in many countries. However, extant research about excessive use of the Internet is focusing on adolescents and younger adults. There are few studies to explore the topic of the elderly's Internet addiction. The purpose of this study is to investigate the relationship between real-life social support and Internet addiction among older adults during the COVID-19 pandemic. This article adopted a self-reported questionnaire *via* internet links to collect data. A total of 303 valid samples about Internet addiction for the elderly were obtained in China. The results suggested that real-life social support is significantly and negatively related to Internet addiction among the aged. Moreover, the findings revealed that real-life social support could mitigate Internet addiction by increasing the levels of hopefulness and decreasing the feeling of loneliness. We expect that this study can enrich the understanding of the problematic Internet usage within older populations. Finally, the contributions, practical significance, and limitations of this study were discussed.

## Introduction

Internet usage in China has increased significantly in recent years. A report from China Internet Network Information Center suggested that by June 2021, the number of netizens in China exceeded 1 billion. And the number of aged 60 and above was about 123 million, accounting for 12.2% of the total number of Internet users and an increase of 1.9% over 10.3% in June 2020. More specifically, a growing number of older netizens begin using the Internet, smart devices, and social network services in China, with the development of information and communication technology and the successful implementation of the aging strategy. However, the wide usage of internet was accompanied by the concern of the excessive use of internet among the elderly. For example, China's Internet Life of the Elderly 2020 report suggests that more than 100,000 are online almost all day, and 0.19% of seniors spend more than 10 h a day online on some apps. Moreover, the average senior user over the age of 60 spends 64.8 min per day on the Internet, and each senior user logs into applications an average of five times per day. Not only that, but a survey from China reported that almost half of the older respondentias will have negative psychological states such as anxiety or uneasiness when smartphones cannot be connected to the network, which are typical of Internet addiction withdrawal reactions (1). As we can see, problematic smartphone use (PSU)/ Internet addiction (IA) is not only limited to young people, but the elderly who are idle at home may also suffer from Internet/ smartphone dependence (2).

However, to our knowledge, very few studies have considered the issue of IA among the elderly, especially netizens who started to be exposed to digital technologies until a later stage of life. That is, the elderly's Internet addiction is a novel issue emerging in the digital age (3), which is worth studying since it is closely related to spending their meaningful and healthy later life (4). Only a handful of studies analyzed the antecedent of addictive use of social media in elderly groups. For instance, Özbek and Karaş (5) proposed that the probability of social media addiction is higher among the older populations with low levels of education and income, living in villages and towns. Moreover, women are vulnerable to reporting higher levels of social media addiction than those men. But social support from family and from a significant other had significant effects on the addictive use of social media among the elderly. Busch et al. (2) conducted a cross-sectional survey and investigated smartphone usage among older adults, and the results revealed social influence, habit, and self-control are strong predictors of problematic smartphone use in the aged 60 years and older. Other studies indicate that perceived social isolation is associated with problematic social media use among older adults (6).

Even though previous literature has revealed the benefits of Internet use by the old and indicated that encouraging older adults to use the internet may help decrease isolation and depression (7), improve the cognitive health (8), and learn about the risk of disease (9), there will be harmful impacts of excessive use of Internet among the elderly. Prior studies have suggested that Internet addiction can damage the mental health and physical health of individuals (10–13). For older adults, their physical functions are in a state of decline, and excessive use of the Internet can cause greater physical and mental harm than other groups, such as causing eye problems, cervical spondylosis, cardiovascular and cerebrovascular diseases, and insomnia (14). In addition, the Internet is full of false information such as rumors, and if deceived, it may also lead to psychological damage such as depression among the elderly (14). A report from China also indicated that Internet addiction not only affects the physical and mental health of the elderly, such as cervical spondylosis, lumbar spondylosis, and emotional changes, but also affects the daily life of the elderly, such as weakening oral expression and vulnerability to Internet fraud (15). Based on the above considerations, older people are in a worse position physically, mentally, and cognitively compared to younger people, we need to pay more attention to Internet addiction in older adults. However, to the best of our knowledge, it is apparent that the field lacks a coherent understanding of how antecedents exert an effect on IA among the aged. Thus, it is necessary to reveal the mediating mechanisms the above factors could induce IA in the elderly, which will give us a real insight into the causes of the IA and contribute to proposing effective intervention strategies.

Remarkably, although researchers have investigated that social support is negatively related to the elderly's IA (5), few studies have focused on the mechanisms of the relationship between social support, especially the real-life social support (RLSP), and IA in senior citizens. RLSP is significant in later life since it is a fundamental need for the older populations to keep in touch with society, cultivate positive emotional experiences, overcome feelings of loneliness, sustain their state of psychological wellbeing, and improve their levels of hope for the future (16, 17). Prior studies suggested that offline social support protects mental health and reduces the likelihood of Internet addiction (18). Then, can RLSP weaken the IA in the elderly? And what is the internal mechanism of this effect? No prior research has investigated these research issues. To address the research gap and provide important practical implications, we focus on the influence of RLSP on IA, and also explore the paths that RLSP indirectly affects IA in the elderly. That is, the mediating role of hopefulness and loneliness, which is beneficial for opening the “black box” of the influence of RLSP on IA.

**Figure 1:**
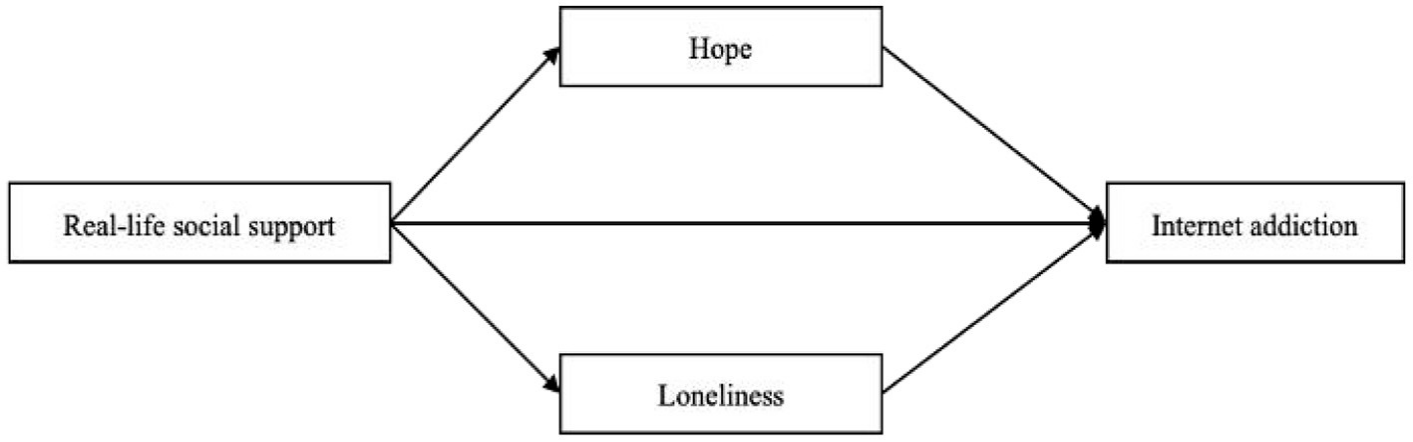
The conceptual model of this study.

Taken together, the principal aim of this study is to explore the relationship between RLSP and IA among those aged 55 years and above during the period of the COVID-19 pandemic. Next, the other purpose of this study is to discuss the mediating role of hope and loneliness in the relationship between RLSP and IA. Figure 1 is the conceptual model.

## Literature review and hypothesis

### Internet addiction

Although numerous studies had been conducted by researchers in the field of Internet addiction over the past decade, there is no clear agreement on which term to use for Internet-use-related problems (19). Diverse terms in the extant studies such as problematic smartphone use, smartphone addiction, excessive Internet use, Internet addiction, Internet dependency, etc. have been adopted and characterized as unsatisfactory Internet use behavior. Meanwhile, prior literature has not formed a unified view about the definition of these terms (20). Young (21) suggested that Internet addiction presented some special characteristics including the inability to stop the desire to use the Internet excessively and the extreme tension felt when losing the Internet. Davis (22) argued that pathological Internet use describes the set of symptoms, i.e., cognitive, emotional, and behavioral symptoms, and cognitive symptoms are prominent among these symptoms, which could induce behavioral symptoms of PIU.

Regardless of the terminology used, they all describe behaviors characterized by symptoms that are similar to those of other addictive behaviors (2). In line with these prior studies, this article adopted the term IA and referred to it as excessive smartphone usage and probably to have difficulty in social or psychological life with five vital symptoms: (1) compulsive use (2) withdrawal symptoms (3) tolerance symptoms (4) interpersonal and health-related problems (5) time management problems. In addition, Internet addiction can cause harm to both individuals and society by preventing people from working or studying (23).

### The relationship between real-life social support and Internet addiction

Social support, a multifaceted concept, is defined as the aid individuals received or perceived from their network members (24). Several opposing types of social support are distinguished in prior literature, i.e., real-life vs. virtual social support (25), visible vs. invisible social support (26, 27), instrumental vs. emotional social support (24). Remarkably, real-life social support, consisting of material assistance and direct services, becomes increasingly vital and plays a protective role in the field of mental disorders such as loneliness and suicidal ideation in the overlapping context of the post-epidemic era and the digital age (28). Particularly, RLSS is a basic but critical need for the elderly during the COVID-19 pandemic, which mainly depends on the following reasons. On the one hand, some evidence has shown that senior citizens are more likely to have a higher rate of COVID-19 infection compared to younger people, they, therefore, could be in a stressful situation where they are concerned about their health. On the other hand, the social range becomes increasingly narrower for the elderly who gradually separated from society as a result of retirement, having a strong need for RLSS. The main sources of RLSS for the elderly are family members and friends. Especially, the frequency of interaction with neighbors and other friends experiences a decreasing trend for older adults under the strict limitation of social distance during the COVID-19 pandemic, and family members become the only source that older people received social support and personal contact. This means that those aged 55 years and older are likely to lack real-life social support.

Compensatory Internet use theory suggests that Internet use is a coping strategy for real-life problems (29). More specifically, the elderly with low levels of RLSS could receive online social support and maintain online relationships *via* using a smartphone or smart network services to fulfill their needs for social support (30). However, upon reviewing the literature, we found this also yielded some results contrary to expected such as problematic smartphone use, and even Internet addiction. Namely, an individual who continually achieves social networks *via* smartphone is more likely to be immersed in a virtual life and forget about the physical world around them, a behavior that may result in Internet addiction (31). Related research has well-documented that the old population with low levels of RLSS shows a higher rate of smartphone addiction (32). For example, Özbek and Karaş (5) argued that elderly individuals who are at risk for lack of RLSS are more likely to turn to excessive use of social media. Meshi and Ellithorpe (18) speculated that problematic social media use was significantly associated with decreased real-life social support and increased social support on social media. In contrast, Real-life social support provides a powerful source in preventing and solving sociological or psychological problems and in dealing with difficult situations (20). The elderly with high levels of RLSS could receive concrete aid and emotional support from the real world, which is more likely to exert an effective function on negative events. That is RLSS may be helpful at the functional level for those aged 55 years older. More specifically, some literature has indicated that RLSS is negatively related to Internet addiction (33).

Therefore, we propose the following hypothesis:

**Hypotheses 1:** Real-life social support is negatively related to Internet addiction among the elderly.

### Hope as a mediator between real-life social support and Internet addiction

Hope is “a positive motivational state that is based on an interactively derived sense of successful (1) agency (goal-directed energy) and (2) pathways (planning to meet goals)” (34). Ersek (35) indicated that hope is “a multidimensional life force that focuses on positive expectations of the future and is always influenced by others.” It is noted that hope for the future is an individual's view and attention to the future and the pursuit of making things better can lead to a sense of social wellbeing, which is a Widely concerned topic in positive psychology.

Hope is an essential need for the elderly (36), and particularly, it was found to be an effective protective mechanism to counteract the negative influences of changes and losses caused by COVID-19. Based on this beneficial effect on the aged, previous research focused on factors that could cultivate and facilitate the level of hope among the elderly. For example, available social support (such as instrumental support from family, and emotional support from friends), as a critical protective factor, contributes to the wellbeing, maintaining positively mental levels, and finding sources of hope for older adults (37). Doolittle and Farrell (38) present that increasing RLSS is more likely to reduce psychological stress and give them more hopefulness when the older population faces the challenge in daily life. High-quality social relationships with their family members and friends could forest a sense of purpose, which is a determinant of promoting hope (39). Taken together, social support was a powerful predictor of hope among the elderly (40). That is, sufficient real-life social support can decrease depression, improve one's general level of happiness and promote the establishment of positive quality, including hope (41).

According to the hope theory, hopefulness focuses on future- and goal-oriented cognition, which could effectively buffer some risky behavior and negative influences. Based on this theory, extant studies have found that hopefulness plays an important role in dealing with psychological distress among individuals who experience some traumatic events (42). Specifically, a person with high levels of hope may better adapt to life challenges, nurture positive psychology, and develop effective strategies when facing additional and tough problems (43–45). Particularly contrasted to the elderly with low levels of hope, hopeful the aged consider their skills to deal with the difficulties and envision a positive future, which may, in turn, lead to lower anxiety and fewer negative outcomes. Following the above logic, older adults with high levels of hope have a set of psychological resources to adequately cope with the Internet usage stress caused by a great deal of fake information and rumor on the Internet during the spread of COVID-19 and the conduction of lockdown. Addiction is a major type of Internet usage stress. Seif et al. (46) also proposed that the elderly full of hope is more likely to have higher self-esteem and adequately cope with the problem of Internet use. Specifically, hope could exert a negative effect on Internet addiction during the COVID-19 pandemic.

Overall, given the positive effect of social support on the hope and the negative role of hopefulness in Internet addiction, we propose the following hypothesis:

**Hypotheses 2:** The relationship between real-life social support and Internet addiction in the elderly is mediated by hope.

### Loneliness as a mediator between real-life social support and Internet addiction

Loneliness has increasingly become a universal human experience, particularly in the digital age (47). There is a consensus that loneliness is a multi-dimensional concept. Researchers have provided various definitions of loneliness in past years. For example, Peplau and Perlman (48) proposed that loneliness is an unpleasant experience, it occurs when there is an incongruence between ideal and achieved personal relationships. Weiss (49) suggested that loneliness consisted of social and emotional aspects. Social loneliness stems from social isolation, whereas emotional loneliness is derived from the deficiency of a close emotional attachment. Although loneliness is distinguished into different types based on various perspectives, it shares a common core that is deficient.

The stress process framework shed light on the that disadvantaged individuals such as the elderly are the most vulnerable to social stressors. We could therefore suggest that those aged 55 and older are more likely to be loneliness compared with other groups, because of many factors, i.e., spousal loss, lack of close friends, and limited social support (50–52). RLSP among the elderly is a critical determinant in the development of successful aging and relieving loneliness. Previous research has revealed the effect of real-life social support for decreasing loneliness in later life among the elderly. For example, Coyle and Dugan (53) imply that the narrowing of social networks contributes to increased social isolation and loneliness. Social isolation is always regarded as a risk factor, while social support can play a protective role in aged later life (54). One possible scenario is that the loss of real-life social support due to the policy of epidemic prevention and control strengthens senior adults' feelings of social isolation and marginality. That is to say, those with higher levels of social network support may have additional material and emotional resources to cope with loneliness.

To reduce loneliness, the elderly is more likely to be attracted to use the Internet for social interaction to strengthen existing friendships and meet their social needs. To be specific, the lonely elderly use video chats or online social media sites to stay in contact with family and friends during the period of lockdown. However, earlier research implies that loneliness increases the risk of experiencing negative outcomes of Internet use (55). Özdemir et al. (19) found that higher loneliness resulted in low self-control, which subsequently led to Internet addiction. Moreover, it was reported that high levels of smartphone addiction were correlated with low self-esteem, loneliness, depression, and shyness (56).

In conclusion, loneliness is associated with Internet addiction (57, 58).

Overall, given the negative effect of real-life social support on loneliness and the positive role of the loneliness in aggravating Internet addiction, we propose the following hypothesis:

**Hypotheses 3**: Loneliness mediates the negative relationship between social support and Internet addiction among the elderly.

## Methods

### Sampling

The data used in this paper were collected *via* a Chinese professional market research company which has built a massive database including numbers of the older population. This database accumulates about 500,000 elderly users in China, as well as their children's relationship network. The market research company selected a sample of 500 people by systematic random sampling according to our needs. Specifically, random sampling was conducted on the basis of taking into account the eastern, central, and western regions in China, as well as the rural elderly and the urban elderly. If it is difficult for the elderly interviewees to fill in the questionnaire, that could be filled in by their children on behalf of the elderly interviewees. Ultimately, 426 of the elderly completed the self-reported questionnaire (response rate = 85.20%). The questionnaire was designed with screening questions, such as attention detection items. Participants who failed to satisfy these screenings would be considered invalid. After removing a number of unqualified ones, we finally obtained 303 valid samples (valid rate = 71.13%), which formed the basis of data analysis in this paper. The survey was collected from January 2022 to February 2022. Participants were given a reward of Renminbi (RMB) 5 when they completed the questionnaire. This fee does not include payments to the research company. Moreover, we did not involve any sensitive topics that may make the participants feel uncomfortable in our questionnaire. All participants were informed of the research process and provided written informed consent in accordance with the Declaration of Helsinki.

A comparison of *t*-test of the valid responding and invalid responding elderly people from the sampling frame indicated no significant differences in terms of key demographic characteristics (i.e., age, gender, education level, and personal income), suggesting that non-response bias may not be a major concern in this research.

In addition, we employed the Heckman two-stage model to assess sample representativeness (59). We recoded the question “How many hours a day do you surf the Internet (the options ranged from 0 to 24 h)” into a dummy variable (i.e., internet access), with 0 h a day coded 0, and others coded 1. We select internet access as the dependent variable in the first stage. We next bring the predicted inverse Mills ratio from the first-stage regression in the second stage of the model. The coefficient of Heckman's lambda is insignificant (β = 0.219, *p* = 0.770), which means that there is no sample selection bias.

### Measurement

#### Real-life social support

We measured the real-1ife social support by the six-item scale from the Multidimensional Scale of Perceived Social Support (MSPSS). Sample items include statements like “I can get emotional help and support from my family when I need it,” and “My friends try to help me.” The items ranged from 1 (strongly disagree) to 5 (strongly agree). All statements on this scale are positive. That means higher scores indicate more social support and lower scores indicate less.

#### Hope

Hope is assessed with the 6 item State Hope Scale developed by Snyder et al. (60). Sample items include statements like “I can think of many ways to reach my current goals” and “At this time, I am meeting the goals that I have set for myself.” Respondents were asked to select the number that best describes how they think about themselves right now. All adopted a five-point Likert scale, ranging from 1 (definitely false) to 5 (definitely true), with higher scores representing higher levels of hope.

#### Loneliness

We used the ULS-8 Loneliness Scale (61), which is a simplified version of the UCLA Loneliness Scale (ULS-20), to assess participants' loneliness. Respondents are asked to indicate the frequency of how often they experience the following “I lack companionship,” “There is no one I can turn to,” “I am an outgoing person” and so on. Each item was measured on a five-point scale (1 = never, 5 = daily). The higher the score was, the greater the loneliness was.

#### Internet addiction

We used the revised Internet Addiction Scale developed by Mak et al. (62) to measure IA among the elderly. The scale of IA comprised a total of 18 items including four dimensions: Compulsive Use of Internet and Withdrawal Symptoms of Internet Addiction (Sym-CW, 6 items), Tolerance Symptoms of Internet Addiction (Sym-T, 4 items), Interpersonal and Health-Related Problems of Internet Addiction (RP-IH, 4 items), and Time Management Problems (RP-TM, 4 items). The respondents are asked to answer the following questions about Internet use according to their practical status. Each item was measured on a five-point scale from 1 (strongly disagree) to 5 (strongly agree). Higher scores indicate a more severe level of Internet addiction.

#### Control variables

To control for alternative explanations, we take demographic information of the participants, including age, gender, education level, personal income, self-rated health, taking care of a grandchild, and living situation. Age was measured in years (range = 55–90), and gender was represented by a dichotomous variable (1 = male, 0 = female). Education level was represented by four categorical variables-primary degrees or below (reference group), secondary degrees, high school degrees, and junior college degrees or above. Self-rated health indicated participants' ratings of their overall health, ranging from 1 (poor) to 5 (excellent). Taking care of a grandchild was represented by a dichotomous variable (1 = yes, 0 = no). Also, the living situation is a dummy variable (1 = living alone, 0 = living with a significant other. The level of individual income per month was represented by four categorical variables: < RMB 1,000 (reference group), RMB 1,000–2,000, RMB 2,000–5,000, and more than RMB 5,000.

### Common method bias tests

Harman's single factor test was conducted to check for the potential effects of common method bias. All scale items were subjected to exploratory factor analysis in SPSS to test for common method variance. The results showed that in the unrotated factor structure, the variance explained was 30.65%, which did not reach 50% (63), so there was no serious common method bias in the sample data.

In addition, we used confirmatory factor analysis to test for common method bias in MPLUS software. We analyzed all scale measures inside a factor, and the results showed a poor model fit (RMSEA = 0.182; CFI = 0.396, TLI = 0.339; SRMR = 0.145), which indicated that all measures should not belong to the same factor (64). Thus, common method bias was unlikely to be of great concern in the sample data. In addition, there was no significant difference between our sample and the non-response sample in terms of age, gender, education level, and *t*-test. Therefore, the effect of non-response bias is very limited (65).

### Analytic strategy

To test our three hypotheses, we implement several quantitative methods, including descriptive statistics, correlations analysis, and multivariable approaches. Multivariable approaches contained OLS regression, confirmatory factor analysis (CFA), and Bootstrap method.

## Results

### Reliability and validity tests

We assessed the reliability and validity of latent variables with confirmatory factor analysis (CFA). First, we ran a CFA model of the four first-order factors of Internet addiction (i.e., Sym-CW, Sym-T, RP-IH, and RP-TM) (see Table 1, Model A). Then we conduct an overall CFA model that includes all latent variables, in which Internet addiction was treated as a second-order factor with four summated indicators of the first-order factors (see Table 1, Model B).

**Table 1:** The validity and reliability of variables.

**Variables**	**Factor loadings**	**CR**	**AVE**
**A. Four first-order factors of internet addiction** ${\text{χ}}_{\left(126\right)}^{2}$ = **342.411**, ***p***<**0.001; CFI** = **0.949; TLI** = **0.939;** **RMSEA** = **0.075; SRMR** = **0.047**			
Sym-CW	0.814	0.923	0.665
	0.767		
	0.864		
	0.791		
	0.840		
	0.815		
Sym-T	0.860	0.885	0.659
	0.895		
	0.678		
	0.798		
RP-IH	0.768	0.870	0.625
	0.830		
	0.806		
	0.757		
RP-TM	0.828	0.871	0.628
	0.798		
	0.806		
	0.735		
**B. Overall model** ${\text{χ}}_{\left(242\right)}^{2}$ = **585.267**, ***p***<**0.001; CFI** = **0.918; TLI** = **0.906;** **RMSEA** = **0.068; SRMR** = **0.054**			
Real-life social support	0.617	0.863	0.515
	0.709		
	0.753		
	0.632		
	0.797		
	0.776		
Hope	0.735	0.838	0.464
	0.752		
	0.664		
	0.588		
	0.647		
	0.689		
Loneliness	0.709	0.894	0.515
	0.855		
	0.696		
	0.687		
	0.839		
	0.631		
	0.638		
	0.651		
Internet addiction	0.743	0.881	0.653
	0.678		
	0.935		
	0.853		

CR, composite reliability; AVE, average variance extracted.

Based on the fit index of Model A and B (see Table 1), we found that the adjusted measurement models had a good model fit (66). As the Table 1 shows, all composite reliability (CR) values exceeded the 0.70 benchmark (67); all average variance extracted (AVE) values were above the cutoff value of 0.50 (68). Moreover, we compared the square root of the AVE value of each variable with the variables' correlations with other factors to estimate discriminant validity. The square root of the AVE value was greater than the between variable pairs, which supports the criterion for discriminant validity (65). Overall, the measures of key variables contained competent reliability and validity.

### Descriptive analysis

Table 2 shows the result of descriptive statistics about sample demographics and correlations among all variables, including the means, standard deviations, and correlation coefficients. Of the 303 usable samples, there are 149 male and 154 female participants. The average age is 69.1 years old (SD = 7.14). Only 28 participants live alone among 55 years and older, others live with a significant other. With regards to self-rated health, the proportions of those self-rated healthier and those self-rated unhealthier are more than 50% and <10%, respectively. The number of older people with primary degrees or below is the largest, accounting for 42.9%. Also, about 40.92% of the participant could earn RMB 2,000–5,000 per month, but 21.45% income is < RMB 1,000 per month.

**Table 2:** Descriptive and correlations among all variables.

**Variables**	**(1)**	**(2)**	**(3)**	**(4)**	**(5)**	**(6)**	**(7)**	**(8)**	**(9)**	**(10)**	**(11)**
(1) Hope	1										
(2) Loneliness	−0.254^***^	1									
(3) Real-life social support	0.423^***^	−0.375^***^	1								
(4) Internet addiction	−0.239^***^	0.272^***^	−0.307^***^	1							
(5) Age	0.087	−0.095^*^	0.070	−0.303^***^	1						
(6) Gender	0.013	0.021	−0.022	0.015	0.005	1					
(7) Education level	0.148^***^	−0.019	−0.048	0.204^***^	−0.180^***^	0.169^***^	1				
(8) Personal income	0.216^***^	−0.138^**^	0.050	0.134^**^	−0.123^**^	0.128^**^	0.600^***^	1			
(9) Self-rated health	0.251^***^	−0.268^***^	0.133^**^	0.000	−0.087	0.075	0.149^***^	0.196^***^	1		
(10) Taking care of grandchild	0.019	0.048	−0.045	0.138^**^	−0.155^***^	−0.093	0.012	0.039	0.057	1	
(11) Living situation	−0.066	0.138^**^	−0.124^**^	−0.074	0.171^***^	−0.630	−0.116^**^	−0.135^**^	−0.080	−0.111^*^	1
Mean	3.455	2.481	3.862	2.046	69.073	0.492	2.132	2.578	3.601	0.422	0.092
SD	0.595	0.447	0.610	0.757	7.142	0.501	1.146	1.029	0.835	0.495	0.290

N = 303.

* P < 0.1,

** P < 0.05,

*** P < 0.01. Gender: 1-male, 0-female; Education level: 1-primary degrees or below, 2-secondary degrees, 3-high school degrees, 4-associate degrees or above; Ling with children: 1-yes, 0-no; Taking care of grandchildren: 1-yes, 0-no.

Besides, there remains a close relationship among these core variables in the present study. Namely, real-life social support is associated with Internet addiction among the elderly (r = −0.307, *p* < 0.01), loneliness (r = −0.375, *p* < 0.01), and hope (r = 0.423, *p* < 0.01). As we expected, hope is significantly correlated with Internet addiction of the old (r = −0.239, *p* < 0.01) and loneliness is significantly related to the elderly's Internet addiction (r = 0.272, *p* < 0.01) as well. This will help us to understand (1) what is the relationship between real-life social support and Internet addiction in the elderly population? (2) And how does real-life social support affect Internet addiction in older adults?

### Hypothesis testing

We conducted a hierarchical regression to assess the association between the factors in our models. Hypothesis 1 of this study predicts that real-life social support could reduce the probability of Internet addiction among the elderly. As shown in Table 3, RLSS is negatively related to Internet addiction (β = −0.356, *p* < 0.01, Model 3), supporting Hypothesis 1.

**Table 3:** Regression coefficients in the mediation model.

	**Loneliness**	**Hope**	**Internet addiction**			
	**Model 1**	**Model 2**	**Model 3**	**Model 4**	**Model 5**	**Model 6**
**Independent variable**						
Real-life social support	−0.230^***^	0.383^***^	−0.356^***^	−0.282^***^	−0.274^***^	−0.207^***^
**Mediators**						
Loneliness				0.321^***^		0.309^***^
Hope					−0.214^***^	−0.203^***^
**Control variables**						
Age	−0.007^*^	0.009^*^	−0.025^***^	−0.023^***^	−0.023^***^	−0.021^***^
Gender	0.043	−0.023	−0.014	−0.028	−0.019	−0.032
Education level	0.021	0.047	0.079^*^	0.072^*^	0.089^***^	0.082^*^
Personal income	−0.052^*^	0.072^*^	0.031	0.048	0.047	0.062
Self-rated health	−0.117^***^	0.121^***^	−0.014	0.024	0.012	0.047
Taking care of grandchild	0.045	0.045	0.123	0.109	0.133	0.118
Living situation	0.152^*^	0.017	−0.110	−0.159	−0.106	−0.153
Constant	4.285^***^	0.608	4.924^***^	3.549^***^	5.054^***^	3.724^***^
*R* ^2^	0.219	0.259	0.204	0.232	0.225	0.251

N = 303.

* p < 0.1,

*** p < 0.01.

To examine the indirect effects of RLSS on Internet addiction *via* hope and loneliness, we estimated mediated path models. First, the control variables and independent variables are entered into Model 1. The regression result of Model 1 indicates that RLSS has a negative relationship with loneliness (β = −0.230, *p* < 0.01). Second, Model 4 is that we add the mediating variable based on Model 1. The result represents that loneliness is positively correlated with Internet addiction, as RLSS is controlled (β = 0.321, *p* < 0.01). Meanwhile, RLSS is negatively related to Internet addiction (β = −0.282, *p* < 0.01) after controlling loneliness. Therefore, according to the causal steps approach used by (69), we propose that loneliness plays a mediating role in the link between RLSS and IA, supporting Hypothesis 2.

Similarly, RLSS is positively associated with hopefulness (β = 0.383, *p* < 0.01, Model 2), while hopefulness negatively predicted Internet addiction among the older population after RLSS was controlled in the regression model 5 (β = −0.214, *p* < 0.01, Model 5). That is, the indirect effect of RLSS on the elderly's IA *via* hope is significant and negative, which supports Hypothesis 3.

Moreover, we adopted PROCESS macro model template 4 for SPSS 26.0 with 5,000 bias-corrected bootstrap samples and 95% confidence intervals (CIs) to further examine the main and mediating effects of this study. When the 95% confidence interval (CI) does not contain zero, this means the statistical significance is achieved. That is the hypothesis is supported. Results are presented in Table 4. Real-life social support is negatively associated with Internet addiction in the elderly [β = −0.376, SE = 0.066, 95% CI = (-0.486,−0.225), CI does not include zero], and Hypothesis 1 is tested again. Furthermore, the results of the test of the mediation model indicate that hope mediates the relationship between RLSS and Internet addiction [β = −0.084, SE = 0.033, 95% CI = (−0.162, −0.028), CI does not include zero]. In parallel, loneliness plays a mediating role in the association between RLSS and IA [β = −0.085, SE = 0.032, 95% CI = (−0.154, −0.030), CI does not include zero]. Hence, these findings provided effective support for Hypothesis 2 and 3.

**Table 4:** Direct and indirect effects of social support *via* hope and loneliness on internet addiction.

**Paths**	**Effect**	**SE**	**LLCI**	**ULCI**
Total effect	−0.376	0.066	−0.486	−0.225
**Direct path**				
RLSP→ IA	−0.207	0.074	−0.352	−0.062
**Indirect path**				
RLSP→ Hope→ IA	−0.084	0.033	−0.162	−0.028
RLSP→ Loneliness→ IA	−0.085	0.032	−0.154	−0.030

RLSP, real-life social support; IA, Internet addiction. Bootstraps resample = 5,000, Control variables are included in the analysis.

## Discussion

With the further promoting of information technology and strictly controlling of COVID-19, the increasingly older population engages in online entertainment instead of offline activities in China. Although a large number of studies focus on the negative effects of Internet use or smartphone use among the elderly, e.g., inducing depression and reducing subjective wellbeing (70), few studies investigate the problem of Internet addiction for the aged 55 years and older. It is important to consider the ramifications of Internet abuse for senior citizens, as the Internet has rapidly become an essential tool in their daily life. To fill this gap, this study indicates the significance of real-life social support during the period of prevention and control of the COVID-19 epidemic and attempts to reveal the underlying mechanism in the relationship between RLSS and IA among senior citizens. Specifically, this article examined a theoretical model that demonstrated the mediating role of hope and loneliness in the relationship between real-life social support and Internet addiction.

This study yields several notable findings regarding the link between RLSS and IA among adults aged 55 years and older. The present study initially revealed that real-life social support is negatively related to Internet addiction for the elderly. Specifically, older adults with higher levels of real-life social support might decrease the probability of Internet addiction during the COVID-19 pandemic. This finding is congruent with the prior literature, which has found social support to be a strong indicator of Internet addiction behaviors (71). One possible scenario is that higher RLSS could effectively meet the social and emotional needs of the elderly, and help the aged affirm and express their identity, accordingly, affecting their physical and psychological health positively. Virtually, the older population is required to abide by coronavirus rules that stay home as much as possible and avoid physical contact with neighbors and strangers for reducing the chance of infection when the COVID-19 pandemic is in China. Accordingly, the frequency of interaction with neighbors and friends showed a dramatically decreased trend, especially for the senior adults with lower RLSS. They thereby have to seek and obtain alternative resources and support *via* online interaction, which will intensify their dependence on smart devices and pose a threat of Internet addiction.

The findings also present that hope mediates the relationship between RLSS and IA among senior citizens. More specifically, this result suggests that the aged with high levels of social support from their family members and close friends will have a promising prospect for the retired life, which extremely reduces the chance of Internet addiction. One possible reason for this finding is that RLSS is easily perceived and visible, which could help the elderly to protect against psychological distress and attain some coping strategies. Especially, they might obtain a large amount of information, resources, and emotional support from their family and friends, consequently, focusing on how to improve the quality of individual life instead of social media exposure frequently during the period of the COVID-19 pandemic.

Furthermore, the results of this study reveal another mediating mechanism of how RLSS exerts an effect on Internet addiction among the aged. That is loneliness plays a mediating role in the association between RLSS and IA. RLSS provides powerful sources for preventing and solving psychological problems, such as loneliness (54), while the feelings of loneliness exert a catalytic effect on Internet addiction. To our knowledge, this study is earlier to investigate the mediating role of loneliness in the relationship between RLSS and Internet addiction.

It is also interesting that the findings suggest age significantly and negatively predicts the level of IA. Compared with the oldest old, younger elderly are excelled at utilizing the smart devices and various phone applications. Therefore, there is a real possibility to use the smartphone excessively. In addition, the education level has a positive relationship with IA for those 55 years and older. A possible explanation for this could be that the aged with a higher degree have reserved funds of knowledge and have a strong learning capacity. They thereby could adapt and integrate into the digital society, and show a narrower digital divide, which also enhances the probability of Internet addiction than low education counterparts. Taken together, Internet addiction is also a differentiated problem within the older age group. However, this variation in IA among the elderly is rarely explored in existing research.

## Practical implications

From a practical perspective, the findings of our study have important implications for both the field of aging study and the digital society.

First, we should not unilaterally suppose that only adolescents is likely to encounter a problem of Internet use such as addictive behaviors. It is more likely to be a prevalent problem among senior citizens with the development of successful aging and information technology. The negative effect of “Internet addiction on the elderly tends to be more terrible compared to younger.” One possible explanation is that older adults, as the disadvantaged cohort, are prone to experiencing psychological trouble and fall into loneliness during the prevention and control of the COVID-19. They are more likely to increase the frequency of social media use to relieve feelings of loneliness, inducing the problem of Internet addiction. Meanwhile, the elderly have difficulty independently addressing the problem behavior such as Internet addiction as a result of a lack of digital literacy. Therefore, it is necessary to develop digital literacy and addiction prevention programs should aim to inform users about the risks associated with excessive SNS use (72, 73).

Second, this study reveals that real-life social support plays a crucial role in preventing problem behavior such as Internet addiction. Real-life social support is a major resource associated with wellbeing in the face of stress (74). This is especially important for older people who are disadvantaged and the most vulnerable to social stressors (75). However, it is a common phenomenon that individuals anywhere (i.e., in restaurants, coffee houses, etc.) are absorbed in using their smart devices in real life, instead of interacting face-to-face with each other (71). Taken together, we argue that older adults should draw attention to the importance of participating in social activities in an offline environment, which is more likely to facilitate the social relationships with their friends, reducing their dependence on smart devices (33). In addition, family is still the fundamental source of safety. Thus, the quality of relationships formed with the family is expected to be effective in gaining social support.

Third, this research also reveals that hope and loneliness play importantly mediating roles in the relationships between real-life social support and Internet addiction among the elderly. Thus, the community may target the aged to help them increase positive psychological experience and foster hopefulness (76). For instance, interventions should be focused on developing some positive coping skills for social distance during the period of pandemic prevention and control (e.g., doing exercise regularly at home, getting some sunlight, reducing social media exposure, etc.). Furthermore, it is an effective approach to giving emotional support to older people to enhance their positive psychological quality, including hope.

## Limitations and future research

This study also presents some limitations. First, our participants all come from China and the study is not national representative, which to some extent influences our findings' generalizability. Thus, our findings are limited in terms of external validity and universality. However, it is true that the digital level has presented a significantly increasing trend in China and a large number of retired older adults have been exposed to digital services. Moreover, prior reports and studies have suggested the existence of the elderly's Internet addiction (1). Thus, we argue that our sample is a good and typical case for studying the association between real-life social support and Internet addiction among the elderly. Future research should pay more attention to the Internet addiction of the aged 55 years and older in other contexts such as developed countries where they rank highly on digitalization indexes or the country where the rate of aging is growing faster. And future research could be national representative to conduct empirical tests, which helps to provide more rigorous evidence for the external validity and universality of the research conclusions.

Second, we take all samples as the object of study and do not make a further distinction as a result of the relatively small sample. As is well known, there are large differences among the elderly. For example, whether the negative relationship between social support and the Internet is different in the aged group having various social and economic statuses. Hence, future research should adopt a perspective of comparative analysis and focus on the heterogeneity of Internet addiction among the elderly. Moreover, future research wants to delve deeper into the results by using a bigger sample size.

Thirdly, social support is a multidimensional concept that is generally defined as the tangible or intangible resources that individuals receive from their social network (77). We principally employed offline social support to measure the perceived social support in the aged 55 years and older in the present study. But what we cannot ignore is that online social support in the digital age is increasingly crucial for the elderly during the period of the COVID-19 pandemic (78). We are uncertain whether the relationship between the various dimensions of social support and Internet addiction presents great contrasts. These constitute new avenues of research with a promising future in this field of study.

Fourth, since this paper is a cross-section study of questionnaire data, it does inevitably exist endogeneity issue. Thus, the lack of examination of the endogeneity problem is a shortcoming of our paper. Future research could test and control the endogeneity problem by longitudinal tracking survey or instrumental variable method.

## Conclusion

Although numerous older people already spend much time on the Internet and have shown some features of Internet addiction during the period of the COVID-19 pandemic, we have had little knowledge of the relationship between real-life social support and the Internet addiction, and also, there is few evidence to reveal the underlying mechanisms that real-life social support could exert effects on the Internet addiction among the senior adults. This article shows that real-life social support is a vital driver of Internet addiction among older adults during the period of the COVID-19 pandemic. Moreover, this study presents a theoretical framework to better clarify that hope and loneliness play a mediating role in the relationship between real-life social support and Internet addiction. We hope our findings could encourage researchers to pay attention to the problematic Internet use among the aged groups in the digital and aging era and inspire practitioners to design more effective settings to reduce online time and the possibility of Internet addiction among those aged 55 and older.

## Data availability statement

The datasets presented in this article are not readily available due to confidentiality requirements. Queries regarding the datasets should be directed to yangyangwhu@whu.edu.cn.

## Ethics statement

The studies involving human participants were reviewed and approved by School of Sociology, Wuhan University. Written informed consent for participation was not required for this study in accordance with the national legislation and the institutional requirements.

## Author contributions

YJ contributed to writing original draft, conceptualization, data curation, formal analysis, and methodology. TL contributed to resources, data collection, and supervision of the article. YY contributed to data curation, methodology, review, and editing. All authors contributed to the article and approved the submitted version.

## Funding

This work was supported by the National Natural Science Foundation of China [72102170 and 72172107] and Independent Research Project of Wuhan University [2021XWZY009].

## Conflict of interest

The authors declare that the research was conducted in the absence of any commercial or financial relationships that could be construed as a potential conflict of interest.

## Publisher's note

All claims expressed in this article are solely those of the authors and do not necessarily represent those of their affiliated organizations, or those of the publisher, the editors and the reviewers. Any product that may be evaluated in this article, or claim that may be made by its manufacturer, is not guaranteed or endorsed by the publisher.
